# 2-(1,3-Benzoxazol-2-yl)-1-phenyl­ethenyl benzoate

**DOI:** 10.1107/S1600536811036920

**Published:** 2011-09-30

**Authors:** Mohammad Hassan Ghorbani

**Affiliations:** a Falavarjan Branch, Islamic Azad University, Falavarjan, Isfahan, Iran

## Abstract

In the title mol­ecule, C_22_H_15_NO_3_, the configuration about the ethyl­enic double bond is *Z* configuration and it is approximately coplanar with the adjacent phenyl ring and benzoxazole ring system as indicated by the C(H)=C(O)—C_phen­yl_—C_phen­yl_ and O_benzoxazole_—C—C(H)=C(O) torsion angles of 179.88 (15) and 5.7 (2)°, respectively. The dihedral angle between the essentially planar (r.m.s. deviation = 0.080 Å) 2-(1,3-benzoxazol-2-yl)-1-phenyl­ethenyl group and the benzoate phenyl ring is 61.51 (6)°. A short intra­molecular O⋯O non-bonded inter­action of 2.651 (2) Å is present.

## Related literature

For background and synthetic details, see: Ciurdaru & Ciuciu (1979 ▶); Zhou & Pittman (2004 ▶). For related structures, see: Markham *et al.* (1999 ▶); Punte *et al.* (1990 ▶); Loghmani *et al.* (2007) ▶. For van der Waals radii, see: Bondi (1964 ▶).
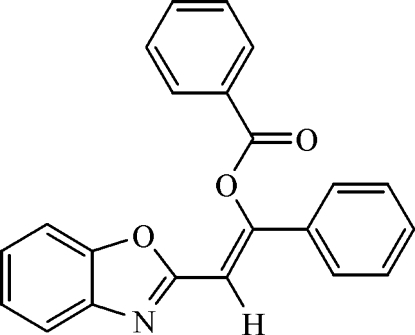


## Experimental

### 

#### Crystal data

C_22_H_15_NO_3_
*M* *_r_* = 341.35
Monoclinic, 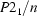

*a* = 10.0152 (11) Å
*b* = 13.1911 (15) Å
*c* = 13.4430 (15) Å
β = 110.957 (2)°
*V* = 1658.5 (3) Å^3^
*Z* = 4
Mo *K*α radiation
μ = 0.09 mm^−1^
*T* = 150 K
0.30 × 0.30 × 0.20 mm

#### Data collection

Bruker SMART 1K CCD diffractometer
Absorption correction: multi-scan (*SADABS*; Bruker, 2007) ▶ *T*_min_ = 0.973, *T*_max_ = 0.982
10417 measured reflections
3254 independent reflections
2656 reflections with *I* > 2σ(*I*)
*R*_int_ = 0.021

#### Refinement

*R*[*F* ^2^ > 2σ(*F* ^2^)] = 0.037
*wR*(*F* ^2^) = 0.093
*S* = 1.08
3254 reflections
236 parameters
H-atom parameters constrained
Δρ_max_ = 0.21 e Å^−3^
Δρ_min_ = −0.17 e Å^−3^

### 

Data collection: *SMART* (Bruker, 2007 ▶); cell refinement: *SAINT* (Bruker, 2007 ▶); data reduction: *SAINT*; program(s) used to solve structure: *SHELXTL* (Sheldrick, 2008 ▶); program(s) used to refine structure: *SHELXTL*; molecular graphics: *Mercury* (Macrae *et al.*, 2006 ▶); software used to prepare material for publication: *SHELXTL*.

## Supplementary Material

Crystal structure: contains datablock(s) I, global. DOI: 10.1107/S1600536811036920/lh5319sup1.cif

Structure factors: contains datablock(s) I. DOI: 10.1107/S1600536811036920/lh5319Isup2.hkl

Supplementary material file. DOI: 10.1107/S1600536811036920/lh5319Isup3.cml

Additional supplementary materials:  crystallographic information; 3D view; checkCIF report

## Figures and Tables

**Fig. 1.**
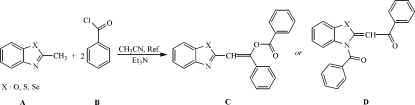
Reactions of 2-methylbenzoazoles with acyl chlorides (benzoyl chloride) in the presence Et3N under reflux conditions and probable products.

**Fig. 2.**
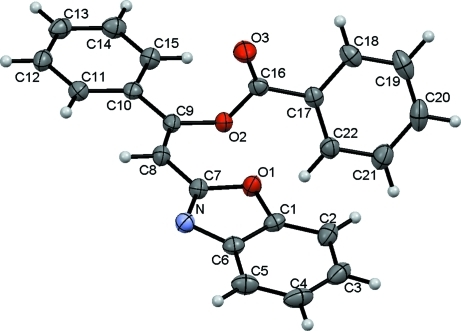
The molecular structure of the title compound, showing 50% probability displacement.

## supplementary crystallographic information

### Comment

The reaction of 2-methylbenzoxazole (A) (Fig. 1) with acyl chlorides such as benzoyl chloride was carried out for the first time by Ciurdaru and Ciuciu (1979), who used the same conditions for reactions of other 2-methylbenzoazoles with acyl chlorides. After infra red and mass spectral investigations and elemental analysis of acylated derivatives, it was suggested that the double acylated structure of (C) was the product of these reactions. This structure differs with the double acylated structure of (D) for the product of acylation of some azoles such as 2-methylthiazoles (Zhou & Pittman, 2004), although these reactions have been done under the same conditions. In fact, based on the presented data, not only the enolester (C) but also the conjugated ketone (D) can be considered as product of the reactions of 2-methylbenzoazoles with acyl chlorides. In addition to the molecular structure, the configuration of the ethylenic double bond in both probable structures was also questionable. In order to clarify these ambiguous situations, the crystal structure determination of the title compound was carried out.

The molecular structure of the title compound is shown in Fig. 2. The enolester structure is confirmed as product of the reaction and the ethylenic double bond (C8═C9) has a *Z* configuration. The ethylenic double bond is co-planar with the connected phenyl ring (the torsion angle of C8—C9—C10—C15 is 179.88 (15)°) and also is approximately co-planar with the planar benzoxazole rings (the torsion angles of O1—C7—C8—C9 and N—C7—C8—C9 are equal to 5.7 (2)° and -174.08 (15)°, respectively). On the other hand, the benzoyl moiety is stituated out of the plane of co-planar components (the torsion angles of C16—O2—C9—C8 and C16—O2—C9—C10 are -89.17 (16) and 95.31 (14), respectively).

The C8—C9 bond length (1.335 (2) Å) in the structure is within the normal range of an unconjugated ethylenic double bond. Also, due to the more resonance interaction of nonbonding electrons on the O2 with the π system of C=O relative to C=C, the O2—C16 bond length (1.3641 (17) Å) is shorter than O2—C9 (1.4010 (16) Å). These values may indicate that the π system of ethylenic double bond is not fully delocalized.

An intramolecular O1···O2 non-bonded distance (2.651 (2) Å) is shorter than the sum of the corresponding van der Waals radii (3.04 Å) (Bondi, 1964). This phenomenon is similar to the intramolecular non-bonded interactions between an oxygen atom and atoms of group VIA in the periodic table  (S, Se and Te) (Markham *et al.*, 1999) and shows that an attractive non-bonded interaction between O1 and O2 must be present in the molecule (Punte *et al.*, 1990). This attraction may be responsible for the *Z* configuration becoming the preferred configuration for the ethylenic double bond (Loghmani *et al.*, 2007).

### Experimental

The title compound was prepared as in the literature (Ciurdaru & Ciuciu, 1979), except that benzoyl chloride and triethylamine (both 30 mmol) were used to complete the reaction. Suitable single crystals for X-ray analysis were obtained from an ethanol solution of the title compound at room temperature.

### Refinement

All H-atoms were positioned geometrically and refined using a riding model with C—H distances = 0.95 Å (both aryl and vinyl-H) and isotropic displacement parameters for these atoms were *U*_iso_(H) = 1.2*U*_eq_(C).

### Crystal data

**Table:**

C_22_H_15_NO_3_	*F*(000) = 712
*M**_r_* = 341.35	*D*_x_ = 1.367 Mg m^−^^3^
Monoclinic, *P*2_1_/*n*	Mo *K*α radiation, λ = 0.71073 Å
Hall symbol: -P 2yn	Cell parameters from 6385 reflections
*a* = 10.0152 (11) Å	θ = 2.2–28.2°
*b* = 13.1911 (15) Å	µ = 0.09 mm^−^^1^
*c* = 13.4430 (15) Å	*T* = 150 K
β = 110.957 (2)°	Block, colourless
*V* = 1658.5 (3) Å^3^	0.30 × 0.30 × 0.20 mm
*Z* = 4	

### Data collection

**Table:**

Bruker SMART 1K CCD diffractometer	3254 independent reflections
Radiation source: sealed tube	2656 reflections with *I* > 2σ(*I*)
graphite	*R*_int_ = 0.021
thin–slice ω scans	θ_max_ = 26.0°, θ_min_ = 2.2°
Absorption correction: multi-scan (*SADABS*; Bruker, 2007)	*h* = −12→12
*T*_min_ = 0.973, *T*_max_ = 0.982	*k* = −16→16
10417 measured reflections	*l* = −14→16

### Refinement

**Table:**

Refinement on *F*^2^	Secondary atom site location: difference Fourier map
Least-squares matrix: full	Hydrogen site location: inferred from neighbouring sites
*R*[*F*^2^ > 2σ(*F*^2^)] = 0.037	H-atom parameters constrained
*wR*(*F*^2^) = 0.093	*w* = 1/[σ^2^(*F*_o_^2^) + (0.034*P*)^2^ + 0.6798*P*] where *P* = (*F*_o_^2^ + 2*F*_c_^2^)/3
*S* = 1.08	(Δ/σ)_max_ < 0.001
3254 reflections	Δρ_max_ = 0.21 e Å^−^^3^
236 parameters	Δρ_min_ = −0.17 e Å^−^^3^
0 restraints	Extinction correction: *SHELXTL* (Sheldrick, 2008), Fc^*^=kFc[1+0.001xFc^2^λ^3^/sin(2θ)]^-1/4^
Primary atom site location: structure-invariant direct methods	Extinction coefficient: 0.0063 (8)

### Special details

**Table:**

Geometry. All e.s.d.'s (except the e.s.d. in the dihedral angle between two l.s. planes) are estimated using the full covariance matrix. The cell e.s.d.'s are taken into account individually in the estimation of e.s.d.'s in distances, angles and torsion angles; correlations between e.s.d.'s in cell parameters are only used when they are defined by crystal symmetry. An approximate (isotropic) treatment of cell e.s.d.'s is used for estimating e.s.d.'s involving l.s. planes.
Refinement. Refinement of *F*^2^ against ALL reflections. The weighted *R*-factor *wR* and goodness of fit *S* are based on *F*^2^, conventional *R*-factors *R* are based on *F*, with *F* set to zero for negative *F*^2^. The threshold expression of *F*^2^ > σ(*F*^2^) is used only for calculating *R*-factors(gt) *etc*. and is not relevant to the choice of reflections for refinement. *R*-factors based on *F*^2^ are statistically about twice as large as those based on *F*, and *R*- factors based on ALL data will be even larger.

### Fractional atomic coordinates and isotropic or equivalent isotropic displacement parameters (Å^2^)

**Table:**

	*x*	*y*	*z*	*U*_iso_*/*U*_eq_	
O1	0.52934 (10)	0.29679 (7)	0.67880 (8)	0.0276 (2)	
O2	0.67141 (10)	0.39020 (7)	0.57269 (8)	0.0242 (2)	
O3	0.87424 (12)	0.36358 (9)	0.71372 (9)	0.0385 (3)	
N	0.40724 (13)	0.39634 (9)	0.75280 (10)	0.0275 (3)	
C1	0.44901 (15)	0.23434 (11)	0.71744 (11)	0.0256 (3)	
C2	0.44378 (17)	0.12991 (11)	0.71550 (13)	0.0330 (4)	
H2A	0.4990	0.0902	0.6855	0.040*	
C3	0.35225 (17)	0.08696 (13)	0.76035 (14)	0.0386 (4)	
H3A	0.3434	0.0153	0.7609	0.046*	
C4	0.27291 (17)	0.14641 (14)	0.80472 (14)	0.0397 (4)	
H4A	0.2107	0.1140	0.8341	0.048*	
C5	0.28183 (16)	0.25104 (13)	0.80738 (13)	0.0353 (4)	
H5A	0.2282	0.2910	0.8385	0.042*	
C6	0.37305 (15)	0.29498 (11)	0.76227 (12)	0.0266 (3)	
C7	0.49753 (15)	0.39237 (11)	0.70353 (12)	0.0260 (3)	
C8	0.56266 (15)	0.47952 (11)	0.67458 (12)	0.0266 (3)	
H8A	0.5435	0.5432	0.6997	0.032*	
C9	0.64666 (14)	0.48137 (10)	0.61670 (11)	0.0239 (3)	
C10	0.70965 (14)	0.57141 (11)	0.58589 (11)	0.0242 (3)	
C11	0.68438 (15)	0.66894 (11)	0.61587 (12)	0.0274 (3)	
H11A	0.6263	0.6778	0.6577	0.033*	
C12	0.74315 (16)	0.75261 (11)	0.58514 (13)	0.0315 (4)	
H12A	0.7244	0.8185	0.6055	0.038*	
C13	0.82906 (16)	0.74112 (12)	0.52499 (13)	0.0331 (4)	
H13A	0.8697	0.7988	0.5044	0.040*	
C14	0.85531 (17)	0.64518 (12)	0.49504 (13)	0.0328 (4)	
H14A	0.9143	0.6369	0.4538	0.039*	
C15	0.79595 (16)	0.56091 (11)	0.52499 (12)	0.0288 (3)	
H15A	0.8143	0.4953	0.5038	0.035*	
C16	0.78482 (15)	0.33301 (11)	0.63327 (12)	0.0251 (3)	
C17	0.78117 (15)	0.23113 (11)	0.58611 (11)	0.0246 (3)	
C18	0.90826 (17)	0.17707 (12)	0.61258 (13)	0.0313 (3)	
H18A	0.9954	0.2064	0.6581	0.038*	
C19	0.90733 (19)	0.08035 (13)	0.57236 (14)	0.0392 (4)	
H19A	0.9942	0.0435	0.5895	0.047*	
C20	0.7804 (2)	0.03741 (12)	0.50741 (14)	0.0413 (4)	
H20A	0.7801	−0.0292	0.4805	0.050*	
C21	0.65337 (19)	0.09079 (12)	0.48128 (14)	0.0382 (4)	
H21A	0.5663	0.0608	0.4365	0.046*	
C22	0.65338 (16)	0.18795 (11)	0.52046 (12)	0.0292 (3)	
H22A	0.5665	0.2249	0.5026	0.035*	

### Atomic displacement parameters (Å^2^)

**Table:**

	*U*^11^	*U*^22^	*U*^33^	*U*^12^	*U*^13^	*U*^23^
O1	0.0315 (5)	0.0228 (5)	0.0324 (6)	−0.0026 (4)	0.0162 (5)	−0.0022 (4)
O2	0.0245 (5)	0.0210 (5)	0.0272 (5)	0.0009 (4)	0.0092 (4)	−0.0023 (4)
O3	0.0312 (6)	0.0364 (6)	0.0385 (7)	0.0019 (5)	0.0013 (5)	−0.0067 (5)
N	0.0280 (6)	0.0262 (6)	0.0315 (7)	−0.0010 (5)	0.0145 (6)	−0.0030 (5)
C1	0.0240 (7)	0.0286 (8)	0.0224 (7)	−0.0049 (6)	0.0061 (6)	−0.0003 (6)
C2	0.0375 (8)	0.0263 (8)	0.0321 (8)	−0.0049 (7)	0.0088 (7)	−0.0029 (7)
C3	0.0376 (9)	0.0310 (8)	0.0394 (10)	−0.0105 (7)	0.0041 (7)	0.0052 (7)
C4	0.0296 (8)	0.0492 (10)	0.0369 (9)	−0.0125 (7)	0.0077 (7)	0.0110 (8)
C5	0.0283 (8)	0.0477 (10)	0.0320 (9)	−0.0034 (7)	0.0131 (7)	0.0026 (7)
C6	0.0237 (7)	0.0299 (8)	0.0240 (7)	−0.0015 (6)	0.0058 (6)	−0.0005 (6)
C7	0.0266 (7)	0.0232 (7)	0.0280 (8)	0.0001 (6)	0.0096 (6)	−0.0021 (6)
C8	0.0284 (7)	0.0206 (7)	0.0313 (8)	−0.0004 (6)	0.0116 (6)	−0.0031 (6)
C9	0.0231 (7)	0.0208 (7)	0.0256 (8)	0.0009 (5)	0.0059 (6)	−0.0030 (6)
C10	0.0206 (6)	0.0247 (7)	0.0234 (7)	−0.0003 (6)	0.0032 (6)	0.0003 (6)
C11	0.0255 (7)	0.0255 (7)	0.0297 (8)	0.0004 (6)	0.0082 (6)	−0.0002 (6)
C12	0.0312 (8)	0.0220 (7)	0.0354 (9)	−0.0007 (6)	0.0049 (7)	0.0018 (6)
C13	0.0303 (8)	0.0303 (8)	0.0344 (9)	−0.0050 (6)	0.0062 (7)	0.0085 (7)
C14	0.0308 (8)	0.0372 (9)	0.0322 (9)	−0.0013 (7)	0.0134 (7)	0.0039 (7)
C15	0.0301 (8)	0.0258 (7)	0.0303 (8)	−0.0007 (6)	0.0107 (6)	−0.0009 (6)
C16	0.0227 (7)	0.0265 (7)	0.0284 (8)	0.0000 (6)	0.0119 (6)	0.0018 (6)
C17	0.0286 (7)	0.0236 (7)	0.0256 (8)	0.0028 (6)	0.0147 (6)	0.0033 (6)
C18	0.0321 (8)	0.0341 (8)	0.0300 (8)	0.0080 (7)	0.0139 (7)	0.0062 (7)
C19	0.0500 (10)	0.0355 (9)	0.0383 (10)	0.0206 (8)	0.0236 (8)	0.0109 (8)
C20	0.0667 (12)	0.0214 (7)	0.0448 (10)	0.0061 (8)	0.0312 (9)	0.0011 (7)
C21	0.0468 (10)	0.0288 (8)	0.0433 (10)	−0.0056 (7)	0.0215 (8)	−0.0072 (7)
C22	0.0313 (8)	0.0251 (7)	0.0346 (9)	0.0002 (6)	0.0159 (7)	−0.0021 (6)

### Geometric parameters (Å, °)

**Table:**

O1—C1	1.3764 (17)	C10—C15	1.394 (2)
O1—C7	1.3703 (17)	C11—H11A	0.9500
O2—C9	1.4010 (16)	C11—C12	1.382 (2)
O2—C16	1.3641 (17)	C12—H12A	0.9500
O3—C16	1.2006 (18)	C12—C13	1.384 (2)
N—C6	1.3973 (19)	C13—H13A	0.9500
N—C7	1.2990 (18)	C13—C14	1.381 (2)
C1—C2	1.378 (2)	C14—H14A	0.9500
C1—C6	1.382 (2)	C14—C15	1.387 (2)
C2—H2A	0.9500	C15—H15A	0.9500
C2—C3	1.386 (2)	C16—C17	1.481 (2)
C3—H3A	0.9500	C17—C18	1.390 (2)
C3—C4	1.393 (3)	C17—C22	1.390 (2)
C4—H4A	0.9500	C18—H18A	0.9500
C4—C5	1.383 (2)	C18—C19	1.384 (2)
C5—H5A	0.9500	C19—H19A	0.9500
C5—C6	1.391 (2)	C19—C20	1.380 (3)
C7—C8	1.442 (2)	C20—H20A	0.9500
C8—H8A	0.9500	C20—C21	1.385 (2)
C8—C9	1.335 (2)	C21—H21A	0.9500
C9—C10	1.472 (2)	C21—C22	1.386 (2)
C10—C11	1.398 (2)	C22—H22A	0.9500
			
C1—O1—C7	103.96 (11)	H11A—C11—C12	119.7
C9—O2—C16	117.25 (11)	C11—C12—H12A	119.7
C6—N—C7	104.24 (12)	C11—C12—C13	120.51 (14)
O1—C1—C2	127.91 (14)	H12A—C12—C13	119.7
O1—C1—C6	107.78 (12)	C12—C13—H13A	120.2
C2—C1—C6	124.30 (14)	C12—C13—C14	119.58 (14)
C1—C2—H2A	122.4	H13A—C13—C14	120.2
C1—C2—C3	115.27 (15)	C13—C14—H14A	119.9
H2A—C2—C3	122.4	C13—C14—C15	120.25 (15)
C2—C3—H3A	119.2	H14A—C14—C15	119.9
C2—C3—C4	121.57 (15)	C10—C15—C14	120.75 (14)
H3A—C3—C4	119.2	C10—C15—H15A	119.6
C3—C4—H4A	118.9	C14—C15—H15A	119.6
C3—C4—C5	122.10 (15)	O2—C16—O3	123.06 (13)
H4A—C4—C5	118.9	O2—C16—C17	111.01 (12)
C4—C5—H5A	121.6	O3—C16—C17	125.93 (13)
C4—C5—C6	116.82 (15)	C16—C17—C18	118.42 (14)
H5A—C5—C6	121.6	C16—C17—C22	121.31 (13)
N—C6—C1	108.85 (12)	C18—C17—C22	120.21 (14)
N—C6—C5	131.23 (14)	C17—C18—H18A	120.2
C1—C6—C5	119.92 (14)	C17—C18—C19	119.68 (16)
O1—C7—N	115.18 (12)	H18A—C18—C19	120.2
O1—C7—C8	120.07 (12)	C18—C19—H19A	120.0
N—C7—C8	124.75 (13)	C18—C19—C20	120.08 (15)
C7—C8—H8A	116.1	H19A—C19—C20	120.0
C7—C8—C9	127.73 (14)	C19—C20—H20A	119.8
H8A—C8—C9	116.1	C19—C20—C21	120.43 (15)
O2—C9—C8	118.30 (12)	H20A—C20—C21	119.8
O2—C9—C10	114.55 (12)	C20—C21—H21A	120.0
C8—C9—C10	126.98 (13)	C20—C21—C22	119.92 (16)
C9—C10—C11	121.43 (13)	H21A—C21—C22	120.0
C9—C10—C15	120.22 (13)	C17—C22—C21	119.67 (14)
C11—C10—C15	118.35 (13)	C17—C22—H22A	120.2
C10—C11—H11A	119.7	C21—C22—H22A	120.2
C10—C11—C12	120.57 (14)		
			
C7—O1—C1—C2	−178.43 (15)	O2—C9—C10—C15	−5.06 (19)
C7—O1—C1—C6	0.41 (15)	C8—C9—C10—C11	−0.5 (2)
O1—C1—C2—C3	−179.83 (14)	C8—C9—C10—C15	179.88 (15)
C6—C1—C2—C3	1.5 (2)	C9—C10—C11—C12	−179.26 (13)
C1—C2—C3—C4	−0.5 (2)	C15—C10—C11—C12	0.4 (2)
C2—C3—C4—C5	−0.6 (3)	C10—C11—C12—C13	−0.6 (2)
C3—C4—C5—C6	0.7 (2)	C11—C12—C13—C14	0.4 (2)
O1—C1—C6—N	−0.68 (16)	C12—C13—C14—C15	0.0 (2)
O1—C1—C6—C5	179.74 (13)	C13—C14—C15—C10	−0.3 (2)
C2—C1—C6—N	178.21 (14)	C9—C10—C15—C14	179.68 (14)
C2—C1—C6—C5	−1.4 (2)	C11—C10—C15—C14	0.1 (2)
C4—C5—C6—N	−179.30 (15)	C9—O2—C16—O3	−11.2 (2)
C4—C5—C6—C1	0.2 (2)	C9—O2—C16—C17	169.02 (11)
C7—N—C6—C1	0.66 (16)	O2—C16—C17—C18	157.19 (13)
C7—N—C6—C5	−179.81 (16)	O2—C16—C17—C22	−25.54 (18)
C6—N—C7—O1	−0.42 (17)	O3—C16—C17—C18	−22.6 (2)
C6—N—C7—C8	179.39 (14)	O3—C16—C17—C22	154.63 (15)
C1—O1—C7—N	0.01 (16)	C16—C17—C18—C19	177.96 (14)
C1—O1—C7—C8	−179.80 (13)	C22—C17—C18—C19	0.7 (2)
O1—C7—C8—C9	5.7 (2)	C17—C18—C19—C20	−0.8 (2)
N—C7—C8—C9	−174.08 (15)	C18—C19—C20—C21	0.5 (3)
C7—C8—C9—O2	3.6 (2)	C19—C20—C21—C22	0.0 (3)
C7—C8—C9—C10	178.51 (14)	C20—C21—C22—C17	−0.2 (2)
C16—O2—C9—C8	−89.17 (16)	C16—C17—C22—C21	−177.38 (14)
C16—O2—C9—C10	95.31 (14)	C18—C17—C22—C21	−0.2 (2)
O2—C9—C10—C11	174.55 (12)		
